# A focus reduction neutralization assay for hepatitis C virus neutralizing antibodies

**DOI:** 10.1186/1743-422X-4-35

**Published:** 2007-03-30

**Authors:** Carole Fournier, Gilles Duverlie, Catherine François, Aurelie Schnuriger, Sarah Dedeurwaerder, Etienne Brochot, Dominique Capron, Czeslaw Wychowski, Vincent Thibault, Sandrine Castelain

**Affiliations:** 1 Laboratoire de Virologie, Centre Hospitalo-Universitaire, Amiens, France; 2 Laboratoire de Virologie, CERVI, Hôpital Pitié-Salpêtrière, Paris, France; 3 Service d'Hépatologie, Centre Hospitalo-Universitaire, Amiens, France; 4 Unité d'assemblage et de réplication du virus de l'hépatite C, CNRS-UMR 8161, Institut de Biologie de Lille, Lille, France

## Abstract

**Background/Aim:**

The role of humoral immunity in hepatitis C virus (HCV) infection is poorly understood. Nevertheless, there is increasing interest in characterizing the neutralizing antibodies in the serum of HCV-infected patients. Focus reduction assays have been widely used to evaluate neutralizing antibody responses against a range of non-cytopathic viruses. Based on the recent development of a HCV cell culture system using the genotype 2 JFH-1-strain, we developed a focus reduction assay for HCV-neutralizing antibodies.

**Methods:**

The focus reduction assay was based on a standard microneutralization assay in which immunostained foci on tissue culture plates are counted. The neutralizing anti-HCV antibodies titers of purified serum immunoglobulin samples from seventy-seven individuals were determined using a 50% focus reduction neutralization assay. Each titer was determined as the log value of the reciprocal antibody dilution that reduced the number of viral foci by 50%. IgG antibodies were first purified from each serum in order to avoid the facilitating effect of HDL on HCV entry.

**Results:**

The assay's cut-off using an ELISA and RNA HCV-negative samples was found to be 1.25 log, corresponding to a dilution of 1:18. The assay was compared with a commercial HCV ELISA and exhibited specificity and sensitivity values of 100% and 96.5%, respectively, and good reproducibility (with intra-assay and inter-assay coefficients of variation of 6.7% and 12.6%, respectively). The assay did not show any cross-reactivity with anti-HIV, anti-HBs or heterophile antibody-positive samples. The neutralizing antibodies titers were 2.13 log (1:134) for homologous samples from HCV genotype 2 infected patients harboring the same genotype as JFH-1 and 1.93 log (1:85) for heterologous samples from patients infected by genotypes other than type 2. These results confirm the presence of broadly cross-neutralizing antibodies already reported using the HCV pseudoparticles system.

**Conclusion:**

This study presents a simple, specific and reproducible cell culture-based assay for determination of HCV-neutralizing antibodies in human sera. The assay should be an important tool for gauging the relationship between the neutralizing antibodies response and viral load kinetics in acutely or chronically infected patients and for investigating the possible eradication or prevention of HCV infection by neutralizing antibodies.

## Background

Hepatitis C virus (HCV, a member of the *Flaviviridae *family) is an enveloped, positive-stranded RNA virus that preferentially replicates in hepatocytes. At least 170 million people worldwide are persistently infected with hepatitis C virus. Chronic HCV infection is associated with a significant risk of progression to cirrhosis and hepatocellular carcinoma [1]. Antiviral therapy with pegylated alpha-interferon and ribavirin (the current best therapeutic regimen) is only successful in about 50% of all treated patients.

Better knowledge of the viral and host factors that determine HCV clearance or persistence during the acute stage of infection is needed in order to improve antiviral therapy and to develop efficient vaccines. Studies focusing on innate and cellular immune responses have shown that a sufficiently large HCV inoculum is able to evade, subvert or circumvent the host's defences. At present, the chimpanzee is the only reliable experimental animal model in which the initial post-HCV infection events and the efficacy of vaccine candidates can be evaluated [2]. It has been shown that HCV-specific T-cell immunity is important in the control of HCV infection [3,4]. Several studies have indicated a role for humoral immunity in the acute stage of HCV infection but this aspect remains poorly characterized. The E1 and E2 glycoproteins are thought to be the viral attachment proteins and thus the main targets for HCV-neutralizing antibodies; identification of protective epitopes conserved across different strains of HCV is therefore a major challenge in vaccine design. A number of antibodies capable of blocking E2 binding to cells or cell receptors have been described, [5-8] some of which neutralize HCV entry in animal or cellular models [9,10]. Cell entry has been shown to involve several surface molecules (notably including the tetraspanin CD81 and the SR-BI receptor [11,12]), although further studies are needed to better understand how viral entry occurs and how it might be neutralized. Detection of neutralizing antibodies in human blood had been problematical until an efficient and reliable cell culture system for HCV became available. Hence, the development of an *in vitro *neutralization assay for HCV could be extremely valuable for characterizing the humoral immune response to HCV and for evaluating the potential of passive and active immunization against hepatitis C. Recent studies using an *in vitro *neutralization assay system (based on infectious retroviral pseudoparticles (HCVpp) bearing HCV envelope glycoproteins) have confirmed that HCV-infected patient sera can indeed neutralize infection [13,14]. However, it has also been shown that the neutralizing activity of antibodies from HCV-infected patients is attenuated by a factor present in human serum, identified as the high-density lipoprotein (HDL) fraction [11,13,15]. HDL facilitation of HCVpp entry is a post-binding event [16], suggesting that HDLs favour internalization of virions and thus the latter's escape from neutralizing antibodies.

Recently, an HCV cell culture model (HCVcc) has been developed [17-19], allowing the production of virus particles that can be efficiently propagated in cell culture. Some preliminary neutralization assays have been carried out by these authors. In this study, we describe how we set up a standardized focus reduction neutralization assay based on HCVcc.

## Results

### HCV focus reduction neutralization assay

**Figure 1:**
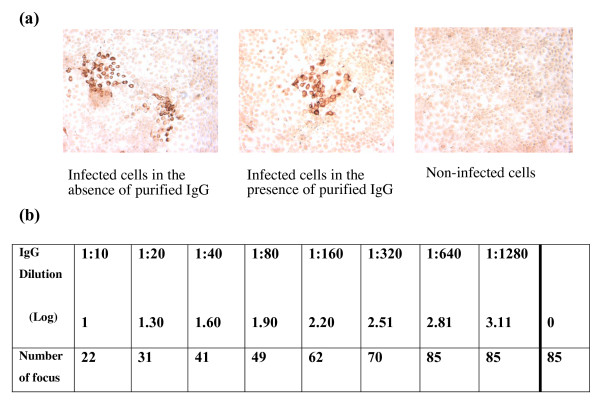
**(a) **Typical pictures of HCVcc-infected Huh-7 cells observed under a light microscope (×40), showing the absence (on the right) and presence (on the left) of focus-forming units (FFU). **(b) **A sample-based neutralizing assay, showing the number of FFU in Huh-7 cells following serial dilutions of purified IgG from a HCV-positive serum sample. Neutralizing anti-HCV antibodies titers were expressed as the highest log dilution of IgG producing a 50% reduction in plaque count, as compared with controls in which the dose of virus was known.

Focus reduction assays have been widely used to evaluate the neutralizing antibody responses to viruses that can form foci in infected cells. Following the recent development of the HCVcc model, the principle of the focus reduction assay has been applied to HCV-neutralizing antibodies detection. The JFH-1 HCV 2a viral strain was grown on a Huh-7 human hepatoma cell line. After three days of infection and cell permeabilization, detection of the HCV foci was carried out using an inactivated HCV-positive patient serum primary antibody and a peroxidase-coupled, Fc-specific anti-human IgG-antibody. The reaction was revealed with DAB peroxidase substrate. The viral foci were thus stained brown, making them easy to count (see Fig. 1a). It has been recently shown that the neutralizing activity of HCV antibodies is attenuated by a serum factor associated with the HDL fraction. Hence, HDLs were able to facilitate HCVpp and HCVcc entry via a mechanism which depended on the expression of the scavenger receptor BI (SR-BI) and its selective lipid-uptake function [11,15,16,20]. In view of the role of HDL in HCV entry, immunoglobulins were purified from each serum sample prior to determination of the neutralizing antibody titer (see Fig. 1b).

### Assay specificity and precision

The specificity of the HCV neutralization assay was assessed by testing 20 anti-HCV-ELISA-negative samples, including five positive for hepatitis B virus surface antibodies (anti-HBs) and five positive for heterophile antibodies. All samples tested negative with two commercial anti-HCV antibody detection assays (Axsym^® ^HCV Version 3.0, Abbott, Wiesbaden, Germany; Vitros^® ^Anti-HCV reagent pack, Ortho-Clinical Diagnostic, High Wycombe, United Kingdom) and HCV-RNA-negative with a qualitative, commercial assay (Cobas Amplicor HCV test Version 2.0, Roche Diagnostics, Meylan, France).

**Figure 2:**
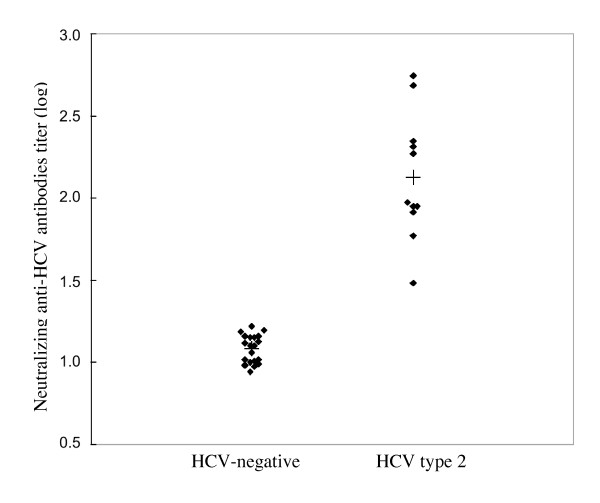
Neutralization of HCVcc JFH-1 in infected Huh-7 cells for two distinct populations of samples, characterized by the presence (HCV genotype 2) or absence (HCV-negative) of anti-HCV antibodies.

These anti-HCV-negative samples were compared with 11 samples from patients chronically infected with HCV genotype 2. The neutralization titers of anti-HCV-negative serum samples are shown in Fig. 2., with a mean value of 1.083 ± 0.083 (corresponding to a dilution of 1:12). The assay's cut-off (determined as the mean value for negative samples plus two standard deviations) corresponded to a dilution of 1:18. The assay exhibited specificity and sensibility values of 100% and 96.5%, respectively. The assay did not show any cross-reactivity with anti-HIV, anti-HBs or heterophile antibody-positive samples (data not shown). Conversely, the chronically HCV genotype 2-positive samples displayed strong reactions, with a mean value of 2.128 ± 0.365 (corresponding to a dilution of 1:134) (p < 0.001).

Inter-assay variability was determined by testing one HCV genotype 2 sample in 10 consecutive experiments (n = 10), whereas intra-assay variability was evaluated by testing the same sample 10 times (n = 10) in the same experiment, whilst running the dilution series. The intra-assay and inter-assay coefficients of variation (CV) of the log neutralization titers were 6.7% and 12.6%, respectively.

### Homologous and heterologous genotype reactivity

**Figure 3:**
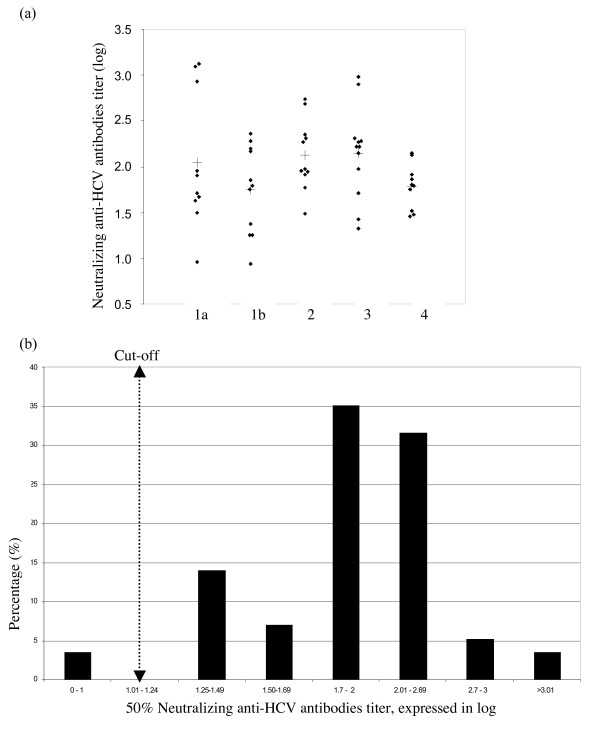
**(a) **Neutralization results for genotypes 1a, 1b, 2, 3 and 4 with respect to HCVcc JFH-1 in infected Huh-7 cells. **(b) **Distribution of the neutralization anti-HCV antibodies titers (independently of the genotype).

Fifty-seven HCV-positive antibodies samples were evaluated using the HCV focus reduction neutralization assay. The genotypes were distributed as follows; for types 1a, 1b, 2, 3, 4 and 5, we studied 11, 11, 11, 12, 10 and 2 samples, respectively. The mean values of the different genotypes is shown in Fig. 3. and Table 1. The mean log neutralization titers for genotypes 1a, 2 and 3 are very similar (2.046 ± 0.671 for genotype 1a, 2.128 ± 0.365 for genotype 2 and 2.148 ± 0.478 for genotype 3). The mean average values are lower for genotype 1b (1.747 ± 0.462) and genotype 4 (1.786 ± 0.236). Strikingly, very high heterologous titers were observed for five patients – three infected with HCV genotype 1a and two infected with HCV genotype 3 (see Fig. 3a). There were too few genotype 5 samples to compare with the other genotypes but the corresponding results nevertheless indicate that the neutralization assay is suitable for this genotype. The two determined log neutralization values for genotype 5 were 1.753 and 1.764, respectively. These results confirm the presence of broadly cross-neutralizing antibodies, as already reported using the previous HCV pseudoparticle system (HCVpp).

**Table 1:** HCV JFH-1 neutralization assay

**Genotype**	**Nb^a^**	**M/F^b ^ratio**	**Age (yr)**	**HCV RNA^c ^(log IU/mL)**	**Mean ± SD^d^**	**Range^e^**
**1a**	11	7/4	43 ± 6	5.90 ± 0.6	2.046 ± 0.671	0.926 – 3.121
**1b**	11	5/6	48 ± 16	6.16 ± 0.5	1.747 ± 0.462	0.934 – 2.356
**2**	11	9/2	63 ± 12	5.50 ± 0.9	2.128 ± 0.365	1.486 – 2.742
**3**	12	8/4	40 ± 11	5.66 ± 0.6	2.148 ± 0.478	1.326 – 2.977
**4**	10	4/6	45 ± 11	6.07 ± 0.7	1.786 ± 0.236	1.453 – 2.152
**5**	2	1/1	66 ± 14	6.06 ± 0.6	1.755 ± 0.005	nd
**all**	**57**	**34/23**	**48 ± 15**	**5.83 ± 0.7**	**1.970 ± 0.491**	**0.934 – 3.121**

^**a **^Number of samples tested.

^**b **^F, female; M, male.

^**c **^HCV RNA measured using the Amplicor HCV Monitor assay, version 2.0 (Roche Diagnostics, Meylan, France) and expressed in log equivalents of IU/mL.

^**d **^Neutralizing anti-HCV antibodies titers expressed in log units; mean ± SD

^**e **^Range of neutralizing anti-HCV antibodies titers, expressed in log units

The distribution of the log neutralization titers across all the HCV ELISA and RNA-positive samples as a function of the HCV genotype is shown in Fig. 3b. More than 60% of the neutralizing antibodies titers fell in the range from 1.7 to 2.69 log titers, corresponding to dilutions of 1:50 and 1:500, respectively. Overall, 3.5% of the samples displayed a titer greater than log 3.0 (1:1000) and, conversely, 3.5% displayed a titer below the cut-off value, i.e. log 1.25 (1:10). Thus, of 57 HCV-infected patients, only two did not test positive for neutralizing antibodies in this assay (the titers were 0.960 and 0.932, respectively).

## Discussion

The role of neutralizing antibodies during acute and chronic viral infection remains an important question and has generated controversial results. Initially, the presence of neutralizing antibodies was shown to control the HCV load and to contribute to viral eradication in patients capable of clearing the infection [13]. In other studies, the appearance of neutralizing antibodies was delayed and restricted to IgG1 antibodies in patients who develop a chronic infection [2,21]. The chimpanzee model has been critical for the study of HCV transmission and host immune responses; however, neutralizing antibodies were not detected in some animals that resolved their infection – suggesting a minimal role in viral clearance, as also observed in human studies [14,15]. Experimentally infected chimpanzees and naturally infected humans can be re-infected with homologous and heterologous HCV strains, suggesting that the humoral immunity that develops after spontaneous resolution of acute hepatitis C is not sterilizing [22-24]. During chronic infection in humans, the presence and/or production of neutralizing antibodies do not suffice for curing the infection but could regulate the spread of the virus. Thus, it can be postulated that during chronic infection, viral mutants can continuously escape the renewed production of neutralizing antibodies.

Retroviral pseudoparticles have been used to develop a very interesting tool for measuring neutralizing antibodies *in vitro *[14]. The assay has demonstrated the presence of HCV-neutralizing antibodies in human sera with relatively high titers (>1:320) and broadly neutralizing activity against different HCV genotypes. However, this model does not represent genuine HCV virions; in particular, the budding of retroviral particles is thought to be very different and may involve a variety of cellular pathways. Characterization of infectious retroviral pseudotype particles bearing HCV glycoproteins have been shown to be very heterogeneous, and so it is possible that these pseudoparticles may not be as relevant as the native HCV virions [25].

The recent development of a cell culture model for HCV enables the production of native HCV virions that can be efficiently propagated in cell culture [17-19]. This cell culture system has allowed us to develop a neutralization assay for evaluating the level and the proportion of HCV-neutralizing antibodies in chronically infected HCV patients. We analysed a number of parameters (such as practicability, reproducibility and specificity) and tested the effect of a range of variables (viral inoculum size, incubation time, fixation and permeabilization methods, blocking and revelation reagents) on these parameters (data not shown). Overall, the neutralization assay described in this study performs similarly to standardized neutralization assays for many other viruses [26-28].

The assay relies on the ability of the specific JFH-1 genotype 2 viral strain to replicate and multiply on a Huh-7 human hepatoma cell line in a cell culture model, enabling the rapid detection of viral foci after 72 hours of infection. Moreover, no secondary foci were detectable at this time point. Fixation with paraformaldehyde and permeabilization with Triton X-100 were chosen in order to preserve antigenicity and prevent the cell monolayer from detaching during washes. Development with DAB peroxide substrate made it easy to count specifically coloured viral foci. The viral inoculum size is an important parameter; it has to be low enough to enable good assay sensitivity but high enough to produce a statistically significant number of foci, i.e. allowing the reduction in the number of foci (and thus the effect of neutralization) to be monitored. Thus, 100 FFUs were used as the inoculum in this neutralization assay.

In order to test different human samples, we had to take into account the ability of HDL to facilitate HCVcc entry via a mechanism which depends on expression of the scavenger receptor BI [11,15,16,20]. Given HDL's role in HCV entry, immunoglobulins were purified from each serum sample prior to determination of the neutralizing antibodies titer; this frees the assay of the risk of non-specific neutralization activity of the serum via the effects of HDL, the complement system and/or serum amyloid A protein (SAA) [29].

The HCV neutralization assay exhibited good reproducibility, for both duplicate assays and independent tests. As expected, the intra-assay coefficient of variation (CV) was lower than the interassay CV. The test also showed good specificity, since there was no interaction with anti-HIV, anti-HBV or heterophile antibodies. Very low titers were found with HCV ELISA and RNA-negative samples, and the assay's cut-off was determined as the mean titer for negative samples plus two standard deviations (1.25 log, corresponding to a dilution of 1:18).

Given that only the JFH-1 strain of HCV genotype 2a was available for the assay, we evaluated the neutralization titer of sera from patients chronically infected with other HCV genotypes, i.e. 1, 2, 3, 4 and 5. Most of these sera were detected as positive by the neutralization assay, except for two sera from HCV genotype 1-infected patients. These two samples presented a high specific antibody ratio according to the ELISA but only very low inhibition by neutralization assay (far below the cut-off, in fact). We conclude that either the samples lacked neutralizing antibodies or that any such antibodies that were present did not cross-neutralize with HCV genotype 2a. The sensitivity was 100% – not only for genotype 2 (the genotype of the strain used for the assay) but also for other HCV genotypes (except genotype 1). HCV genotype 5 antibodies were also measured but there were too few samples for accurate testing. Moreover, the positive sera (96.5%) had comparable and significantly high titers (1.99 ± 0.63), whatever the genotype. This finding suggests that most neutralizing antibodies are cross-reactive. Another possibility is that most of the patients had been previously infected by a genotype 2 strain. However, this is unlikely because few genotype 2 strains are circulating in France [30]. As expected for a neutralization test, the assay presented in the present study appeared to be very specific (independently of the genotype) and usable in most circumstances. For most viral infections, neutralization assays such as that described in this study are used as reference assays. Thus, we are confident that as other HCVcc genotypes become available, these assays will replace the pseudoparticle assay in the near future because they are probably more relevant. Our assay is somewhat time-consuming and could be simplified by using one dilution to count the foci; however, this type of "short cut" would make it difficult to extrapolate to the dilution neutralizing 50% of the inoculum. Another approach would consist in using recombinant HCV capable of expressing reporter genes (such as luciferase) in order to use a single dilution and obtain a quantitative result [31]. However, further neutralization studies using other genotypes are needed in order to complete our observations and to characterize the homologous and heterologous potencies of polyclonal and monoclonal neutralizing antibodies.

## Conclusion

A simple, specific and reproducible cell culture-based neutralization assay was developed for the determination of neutralizing anti-HCV antibodies in human sera. This test should be an important tool for gauging the relationship between the neutralizing response and viral load kinetics in acutely and chronically infected patients.

## Methods

### Cell culture and HCV production

The Huh-7 human hepatoma cells [32] were grown in Dulbecco's minimum essential medium (Invitrogen) supplemented with 10% fetal bovine serum. All cell cultures were maintained in 5% CO_2 _at 37°C.

The plasmid pJFH-1 containing the full-length cDNA of the JFH-1 isolate (which belongs to subtype 2a (GenBank accession no. AB047639)), was a gift from Dr Wakita (Department of Microbiology, Tokyo Metropolitan Institute for Neuroscience, Tokyo, Japan) and has been described previously [17]. To generate genomic HCV RNA, the plasmid pJFH-1 was linearized at the 3' end of the HCV cDNA and used as a template for *in vitro *transcription, as described previously [33]. Viral stocks were obtained by harvesting cell culture supernatants and freezing them at -80°C. Virus titration was performed on Huh-7 cells with 6-well microtiter plates (Corning, NY) 72 hours after incubation, by immunostaining the cells with antibodies from a HCV-positive patient serum that had previously been inactivated at 56°C (see the section on the virus neutralization assay). The viral titer was determined in triplicate from the mean number of foci and expressed as focus forming units/mL (FFU/mL).

### Patients and clinical samples

Seventy-seven human serum samples were tested. Collection of the sera was approved by the local Ethics Committee and informed consent had been obtained from the donors. Fifty-seven of these samples were obtained from chronically infected HCV patients. The presence of HCV antibodies was determined and confirmed using two third-generation HCV EIA assays (Axsym^® ^HCV Version 3.0, Abbott, Wiesbaden, Germany and Vitros^® ^Anti-HCV reagent pack, Ortho-Clinical Diagnostic, High Wycombe, United Kingdom). HCV RNA was determined with a qualitative commercial assay (Cobas Amplicor HCV test Version 2.0, Roche Diagnostics, Meylan, France) and HCV genotyping was performed by direct sequencing, as described elsewhere [34]. The genotypes were distributed as follows: 11, 11, 11, 12, 10 and 2 samples of types 1a, 1b, 2, 3, 4 and 5, respectively. A set of 20 anti-HCV-negative serum samples was used to evaluate the assay's specificity, including five serum samples with positive hepatitis B virus surface antibody (anti-HBs) status and five sera from Epstein-Barr virus-infected patients that had tested positive for heterophile antibodies. All serum samples had been stored at -80°C upon collection and had not been thawed until the time of assay.

### Serum immunoglobulins purification

Serum immunoglobulins G (IgG) fraction was purified using protein G-Sepharose (GE Healthcare, Orsay, France). 400 *μ*L of heat-inactivated serum was mixed with 200 *μ*L of protein G-Sepharose immunobeads for 30 min at 25°C and then centrifuged for 1 min at 3800 g. The supernatant was discarded and the immunobeads were then washed 3 times with 400 *μ*L of Immunopure IgG binding buffer (Pierce, Rockford, USA). 400 *μ*L Immunopure IgG Elution buffer (Pierce, Rockford, USA) were added to the immunobeads, which were mixed thoroughly and then centrifuged for 1 min at 5000 g. The supernatant was pooled and neutralized with 40 *μ*L Tris-HCL 1 M pH 8.0. The IgG concentration was determined using a Bradford assay (Bio-Rad Protein Assay, Bio-Rad, Marnes-la-Coquette, France). Purified IgG was stored at -80°C.

### HCV focus reduction neutralization assay

The HCV focus reduction neutralization assay was performed in 96-well microtiter plates. Serial dilutions of purified IgG (10 *μ*g) ranging from 1:10 to 1:1,280 were established. Each dilution was tested twice. 25 *μ*L of each sample was mixed with 25 *μ*L of virus (100 FFU) in 96-well microtiter plates and incubated for 1 hour at 37°C, 5% CO_2_. A volume of 100 *μ*L of Huh-7 cell suspension (10,000 cells/well) in culture medium was added and incubated for 5 hours at 37°C, 5% CO2. After 5 hours, the supernatants were removed and 100 *μ*L of culture medium were added to the monolayers. After 72 hours, the cells were fixed with paraformaldehyde and permeabilized with 0.5% Triton X-100. Primary antibody (a HCV-positive patient serum inactivated at 56°C) was diluted to 1:500 prior to use and then incubated for 1 h at room temperature. A peroxidase-coupled, Fc-specific anti-human IgG antibody (Sigma, Saint Quentin Fallavier, France) diluted to 1:200 was dispensed onto the cell monolayer and incubated for 30 min at room temperature. The reaction was developed with DAB peroxidase substrate (Sigma, Saint Quentin Fallavier, France) and stopped after 10 min of incubation with distilled water. The number of HCV foci in each dilution was determined. Controls were included in each assay (non-neutralized virus, purified IgG from each patient at a 1:10 dilution). The dilution that neutralized 50% of the virus was calculated by curvilinear regression analysis using XLSTAT 2006 software (Addinsoft SARL, Paris, France) [35]. Each titer was determined as the log value of the reciprocal antibody dilution that reduced the number of viral foci by 50%.

### Statistical analysis

Titers were expressed as logarithmic values and means ± standard deviation were calculated. Student's t-test was used to compare data between groups. p values below 0.05 were considered to be significant.

## Competing interests

The author(s) declare that they have no competing interests.

## Authors' contributions

CFo, SC and GD conceived, designed and performed the analysis. CFo, SD, EB, AS carried out the bioassay. SC and CFr performed the statistical analysis. DC, CW and VT have given final approval for the version to be published. SC and GD wrote the paper.

All authors have read and approved the final manuscript.
